# Rabbit Enteropathies on Commercial Farms in the Iberian Peninsula: Etiological Agents Identified in 2018–2019

**DOI:** 10.3390/ani9121142

**Published:** 2019-12-13

**Authors:** Luis Solans, Jose L. Arnal, Celia Sanz, Alfredo Benito, Gema Chacón, Oihane Alzuguren, Ana B. Fernández

**Affiliations:** Veterinary Diagnostic Disease and Autogenous Vaccine Laboratory, Exopol SL, Poligono Rio Gallego, D/8. 50840 San Mateo de Gallego, Zaragoza, Spain; lsolans@exopol.com (L.S.); jlarnal@exopol.com (J.L.A.); csanz@exopol.com (C.S.); abenito@exopol.com (A.B.); gchacon@exopol.com (G.C.); oalzuguren@exopol.com (O.A.)

**Keywords:** rabbit, digestive, *Escherichia coli*, EPEC, *Clostridium spiroforme*, rotavirus, *Bacteriodes fragilis*, *Eimeria*

## Abstract

**Simple Summary:**

Digestive disorders are the main cause of economic damage in rabbit farms and, usually, antibiotic treatment is the first choice to control them. Nevertheless, a broad range of infectious agents can be involved in such disorders, as we have observed in our diagnosis work as a veterinary diagnostic laboratory. In this study, a global and updated overview of the frequency of detection of those etiological agents is provided. We have seen differences depending on the age of the affected rabbits, with young rabbits (<15 days old) being the most affected by enteropathogenic *Escherichia coli* strains, while in preweaning and growing rabbits, a coinfection of two or three pathogens is the most prevalent situation. *Clostridium spiroforme* and *E. coli* are the main bacterial agents detected in preweaning rabbits, but enterotoxigenic *Bacteroides fragilis* has just appeared as a new possible emergent pathogen. Coinfections between bacteria (*C. spiroforme* and *E. coli*), parasites (*Eimeria* spp.), and viruses (rotavirus) are much more frequent than simple infections in growing rabbits; for this reason, complete laboratory studies are required to establish on-farm disease control measures.

**Abstract:**

Digestive disorders are the main cause of economic damage to rabbit farms. This article provides a global and updated overview of the diverse etiological agents causing them, since 757 clinical cases were analyzed during 2018 and 2019—Ninety-five from young rabbits (<15 days old), 117 from preweaning rabbits (15–35 days old), and 545 from growing rabbits. Etiological diagnosis was carried out by bacteriological culture and a set of real time polymerase chain reaction (qPCR) tests for the detection of enteropathogenic *Escherichia coli* (EPEC), *Clostridium spiroforme*, *C. perfringens*, rotavirus A, *Bacteroides fragilis*, and *Eimeria* spp. Also, 40 EPEC and 38 non EPEC isolates were investigated for the presence of other colonization factors (*afr2*, *ral*, *liftA*, and *paa*) by qPCR. EPEC is the most prevalent agent in young rabbits, and although different virulence profiles have been found among EPEC isolates, the *liftA+*, *ral*+, and *paa*+ profile is the most prevalent. *C. spiroforme* and EPEC are the more frequently detected pathogens in preweaning rabbits, but *B. fragilis* appears to be a new possible emergent pathogen. In growing rabbits, diverse co-infections between *C. spiroforme*, *Eimeria* spp., EPEC, and rotavirus are much more frequent than infections due to only one of them. Other pathogens detected in very few cases are *Salmonella* spp. and *Enterococcus hirae*.

## 1. Introduction

Spain is the largest rabbit (*Oryctolagus cuniculus*) producer of the European Union [1], producing more than 53,000 tons of rabbit meat in 2018, with more than 3800 farms distributed across the entire country [2]. Among the challenges that the rabbit meat industry faces, the most important cause of monetary losses is related to digestive problems; these enteric problems mostly affect young animals, both suckling and growing rabbits, and they are responsible for high morbidity and mortality rates in affected rabbitries, as has been shown in other European countries [3,4]. At Exopol, a Spanish veterinary diagnosis laboratory, 58% of the clinical cases received from rabbit farms throughout 2018 were related to digestive problems. This percentage increased to 79.6% when analyzing growing animals.

Several pathogens have been described as causative agents of enteric disease in rabbits. Enteropathogenic *Escherichia coli* (EPEC) strains are one of the main etiological agents causing these disorders. These EPEC strains can be any serotype or biotype and, moreover, they do not produce known enterotoxins or Shiga toxins; their virulence is associated with intestinal epithelial cell damage by effacing the microvilli and attaching intimately to the cell membrane, producing “attaching and effacing” lesion and diarrhea [5,6,7,8]. Such lesions are due to the locus of enterocyte effacement (LEE) pathogenicity island that codes for the outer membrane protein called intimin (*eae*) among other virulence-associated genes related to the type III secretion system [9]. In addition to the LEE, several colonization factors, encoded by the *afr2*, *ral*, *lifA*, and *paa* genes, have also been found in rabbit EPEC isolates, but their roles remain unclear since there have been EPEC strain isolates lacking these rabbit-specific fimbrial adhesins, suggesting the presence of further potential adhesins that are not yet known [10].

In addition to EPEC, some other bacterial species have been defined as pathogenic to rabbits; one of the most important is *Clostridium spiroforme*. This *Clostridium* produces a binary toxin, similar to the iota toxin from *Clostridium perfringens*, which is considered as the principal virulence factor that causes enterotoxemia in rabbits [11]. It can be found at the late stages of digestive disorders and sometimes has been associated with antibiotic-treatment-related dysbiosis [8]. Another important bacteria-caused disease in rabbits is salmonellosis, which is characterized by metritis in does and septicemia in young rabbits but also causes digestive clinical signs in growing animals [12]. Besides bacteria, parasites also play a pivotal role in enteric disease, with coccidian protozoa being the most important parasites implicated in enteric rabbit diseases, causing considerable economic damage to rabbit farms. Among the rabbit-infecting coccidian protozoa, at least 11 different species of *Eimeria* spp. Have been described, and the severity of the process has been related to the *Eimeria* species implicated and the infection degree [8].

Other etiological agents that have been accepted as potential pathogens, whose presence has been related to several risk factors (such as treatments, inappropriate feeding, or climatic aggressions, among others) and can induce enteric problems, are group A rotavirus [13], *C. perfringens* [14], and *Enterococcus hirae* [15]. More recently, enterotoxigenic *Bacteroides fragilis* has been pointed out as an enteric pathogen that causes severe disorders around the weaning period [16]. Therefore, it is necessary to take into account the possibility of intercurrent agents in an observed disease.

The objective of the present study was to describe the etiological agents identified in samples of the digestive processes of young rabbits (<15 days old), in pre-weaning rabbits (15–35 days old), and in growing-finishing rabbits, sent by veterinarians to our laboratory during 2018 and 2019.

## 2. Materials and Methods

### 2.1. Clinical Cases, Samples, and Isolates Studied

A total number of 757 clinical cases (samples received from an affected farm at an outbreak) coming from 363 different commercial rabbitries were analyzed in this work. We received samples in the laboratory from January 2018 to June 2019 coming from elsewhere in the Iberian Peninsula (Portugal and continental Spain). Received samples were consistent with digestive organs or caecal swabs collected by practitioners from clinically affected growing rabbits. In each clinical case, samples from 3 to 5 animals were analyzed. Of the 757 clinical cases, 95 were due to gastrointestinal disorders in young rabbits (<15 days old), 117 cases were from 15–35 day-old rabbits before weaning, and 545 affected growing rabbits.

Bacteriological cultures and a set of real-time Polymerase Chain Reaction (qPCR) assays for the detection of EPEC, *C. spiroforme*, *C. perfringens*, and Group A rotavirus were carried out in 100% of clinical cases (*n* = 757), whereas *Eimeria* spp. and enterotoxigenic *B. fragilis* were only studied by qPCR in 542 and 349 out of 757 total clinical cases, respectively, as those two latter qPCR tests were included in September 2018 for *Eimeria* spp. and January 2019 for *B. fragilis* in our routine diagnosis service.

The number of clinical cases in which pathogens were analyzed are presented in an age distributed format in Table 1. 

On the other hand 78 EPEC (*n* = 40) and non-EPEC (*n* = 38) *E. coli* isolates obtained by bacteriological culture in this study were selected for the investigation of other colonization factors such as adhesive factor/rabbit 2 (*afr2*), rabbit EPEC adherence locus (ral), linfocite inhibitory factor (*liftA*), and the porcine attaching and effacing-associated (*paa*) gene. 

### 2.2. Bacteriological Culture

Bacteriological culture was carried out individually for each sample. Mucosal digestive samples were obtained by scrapping with cotton swabs, and these were streaked on Columbia blood, McConckey, XLD, and Slanetz and Bartley agar plates (OXOID) and grown at 37 °C under aerobic conditions for 24–48 h. MALDI Biotyper (Bruker Daltonics GmbH, Bremen, Germany) was used to identify bacterial isolates.

### 2.3. Extraction of Total Nucleic Acids from Clinical Samples

For every single case, up to 5 samples were pooled for acid nucleic acid extraction to make only one determination per case and pathogen. The content of swabs used for bacteriological culture was afterwards removed in 700 µL of PBS solution through rotation movement and vortex usage. After that, total nucleic acids were extracted from 200 µL of that suspension using an automatic device (KingFisher Flex System, Thermo Fisher Scientific, Waltham, MA, USA) and the respective commercial kit (MagMAX™ CORE Nucleic Acid Purification Kit, Thermo Fisher Scientific Waltham, MA, USA), following the manufacturer’s instructions.

### 2.4. Extraction of DNA from E. coli Isolates

The DNA from *E. coli* colonies were extracted with a simple thermal shock. The colonies were put in 1 mL of distilled water and boiled for 10 min in a water bath. After that, the tube was centrifuged for 5 min at 15,600× *g*, and the eluted DNA was transferred to a new tube.

### 2.5. Amplification of Nucleic Acids

The above-mentioned pathogens were detected simultaneously from the same DNA elution. Real-time PCR reactions were performed using commercial kits available on the market following the instructions of the manufacturer: EXOone oneMIX *E. coli eae* gene, EXOone oneMIX *C. spiroforme*, EXOone oneMIX *C. perfringens* Alpha Toxin, EXOone oneMIX Rotavirus A, EXOone oneMIX *Bacteroides fragilis*, and EXOone oneMIX *Eimeria* spp. (Exopol, Spain). The gene targets of each commercial kit and their coded proteins are shown in Table 2.

*Escherichia coli* isolates were investigated for the presence of the *eae* gene as well as other colonization factor genes, which are also related to the adherence of the bacteria: *afr2*, *ral*, *lifA*, and *paa*. For that purpose, real-time PCR assays were performed using their respective commercial kits available on the market following the instructions of the manufacturer: EXOone oneMIX E. coli *afr2*, EXOone oneMIX *E. coli ral*, EXOone oneMIX *E. coli lifA*, and EXOone oneMIX *E. coli paa* (Exopol, Spain).

### 2.6. Software Analysis

All graphics were generated using Graphpad Prism 5^®^ software (San Diego, CA, USA), but the Venn diagrams were generated using the online webpage http://www.interactivenn.net/ [17].

## 3. Results

**Figure 1:**
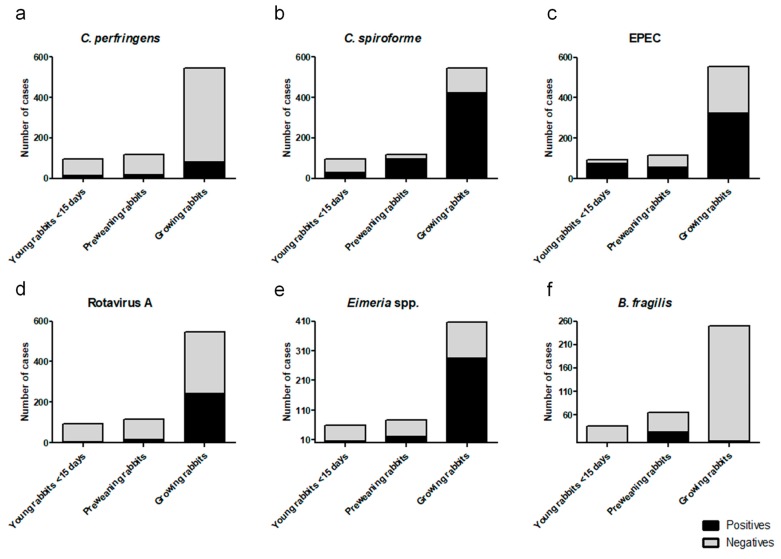
**Rate of detection by qPCR of individual pathogens distributed by age.** The distributions of positive and negative cases within age groups are represented using black for positives and gray for negatives. (**a**) *C. perfringens* analysis where the total number of cases for each group of age was young rabbits (<15 days old), 95; preweaning rabbits, 117; growing rabbits, 545; (**b**) *C. spiroforme* analysis where the total number of cases for each group of age was young rabbits (<15 days old), 95; preweaning rabbits, 117; growing rabbits, 545; (**c**) *EPEC* analysis where the total number of cases for each group of age was young rabbits (<15 days old), 95; preweaning rabbits, 117; growing rabbits, 545; (**d**) *C. perfringens* analysis where the total number of cases for each group of age was young rabbits (<15 days old), 95; preweaning rabbits, 117; growing rabbits, 545; (**e**) *Eimeria* spp. analysis where the total number of cases for each group of age was young rabbits (<15 days old), 59; preweaning rabbits, 77; growing rabbits, 406; (**f**) enterotoxigenic *B. fragilis* analysis where the total number of cases for each group of age was young rabbits (<15 days old), 35; preweaning rabbits, 64; growing rabbits, 205.

Results obtained by qPCR for each of the studied pathogens (*C. perfringens*, *C. spiroforme*, EPEC, rotavirus A, *Eimeria* spp., and enterotoxigenic *B. fragilis*) distributed by age groups are shown in Figure 1.

Regarding microbiological cultures (data not shown), *E. coli* was the most frequently isolated bacteria, but as EPEC positive cases were identified by the *eae* gene-specific qPCR test directly performed on mucosal samples, results of *E. coli* isolation by culture were not taken into account. Only cases with other bacterial species considered to be isolated rabbit pathogens were recorded. *Salmonella* spp. and *E. hirae* were the only pathogens detected by aerobic culture (Table 3). Other bacterial species isolated during this study were *Bacillus* spp., *Proteus* spp., *Staphylococcus* spp., *Enterobacter* spp., *Enterococcus faecalis*, *E. faecium*, *Pseudomonas* spp. *Acinetobacter* spp., and *Alcaligenes* spp., but all of them are considered commensal microorganisms of the digestive tract of rabbits. 

**Figure 2:**
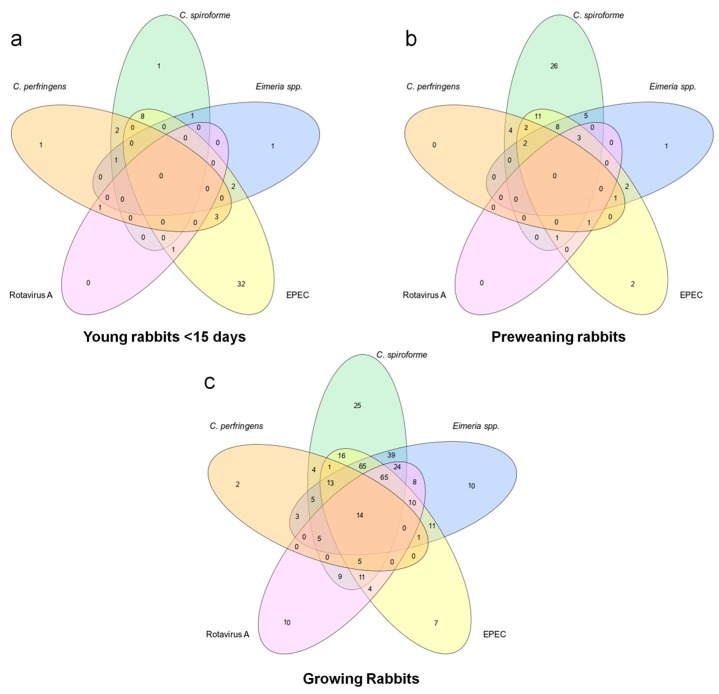
Venn diagrams representing coinfections within positive cases. Positive cases of *C. perfringens* (orange), *C. spiroforme* (green), *Eimeria* spp. (blue) EPEC (yellow), and rotavirus A (pink) are represented in Venn diagrams to illustrate coinfections in the different age groups: (**a**) young rabbits (<15 days old); (**b**) preweaning rabbits; (**c**) growing rabbits.

**Figure 3:**
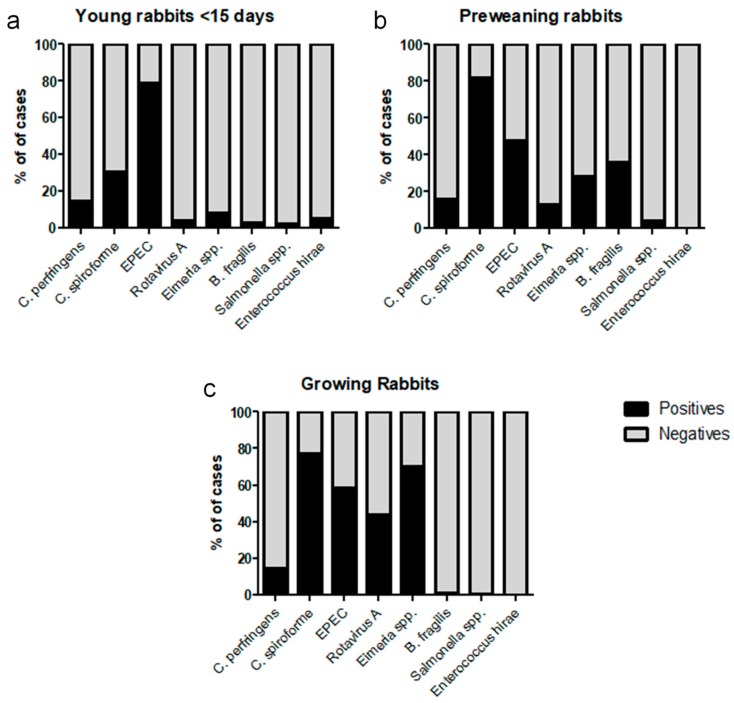
Rate of detection of the rabbit pathogens identified in the present study expressed as percentages and distributed by age. The distributions of positive and negative cases are represented using black for positives and gray for negatives: (**a**) shows 95 young rabbit (<15 days old) clinical cases; (**b**) shows 117 preweaning rabbit clinical cases; (**c**) shows 545 growing rabbit clinical cases.

Figure 2 shows the Venn diagrams representing the principal coinfections found between the cases analyzed by qPCR. Figure 3 shows the proportions of positive and negative cases compared to the total cases analyzed for each of the eight pathogens identified in this study.

The virulence attributes of the 78 *E. coli* isolates studied in this work are listed in Table 4. Non-EPEC isolates (*n* = 38) were also negative for the other colonization factors investigated, whereas all of the EPEC isolates (*n* = 40) were also positive at least to one other colonization factor. Different virulence profiles have been found among EPEC isolates, but the more common one was that positive to *liftA*, *ral*, and *paa* (*n* = 28), while isolates positive for *afr2* were scarce (*n* = 6).

## 4. Discussion

In this work, *C. spiroforme* is the pathogen that is most frequently detected in preweaning and growing rabbits (Figure 3) where it has been found in nearly 80% of the analyzed cases. Although it is mostly found coinfecting the animals with other pathogens, in 41% of the positive cases in the preweaning group, it was the only pathogen identified. In contrast, single *C. spiroforme* infection represented 8% of the total cases of kits <15 days old and growing rabbits (Figure 2). This is consistent with the incidence of *C. spiroforme* in rabbit enteropathies reported in other countries such as France [8], Italy [18], or Canada [19] where it has being usually associated with other pathogens. The high ratio of single *C. spiroforme* infection in preweaning rabbits is an interesting result that might indicate its importance in this age group and should be a matter for further research in the future.

*Clostridium spiroforme* associated enterotoxaemia is not usually detected in very young rabbits; nevertheless, we detected the presence of *C. spiroforme* in 30% of the cases analyzed for the youngest age group (Figure 3). This could be attributed to the high sensitivity of the qPCR used for detection which allowed us to detect the presence of *C. spiroforme* in lower concentrations than the detection limit observed in classic microbiological techniques. It is important to note that, with the current techniques available at Exopol, we can only detect the presence of *C. spiroforme* but not the presence or secretion of its binary toxin into the digestive track. Additionally, *C. spiroforme* can be detected in older animals, and only in determined circumstances can it result in overgrowth and subsequent toxin production leading to enterotoxaemia [20,21]. Factors that can trigger this enterotoxaemia are associated with changes in the immature caecum at weaning [22] and disbalance in the caecal commensal microbiota by the use of antimicrobial drugs [8,18].

Although the frequency of detection of EPEC was lower than *C. spiroforme*, it was detected consistently in the three age groups studied: 79% of positive cases in young rabbits (<15 days old), 48% in preweaning rabbits, and 58% in growing rabbits (Figure 3). The high frequency of EPEC detection in young rabbits is of interest, as it is mostly detected as a single pathogen (Figure 2) and therefore is compatible with neonatal colibacillosis. EPEC was also found in the other age groups; however, it is mainly found coinfecting the digestive track with other pathogens (Figure 2). Colibacillosis is considered as the most important enteric disease in rabbits [7,8,23], and it is associated with the *eae* gene as well as other adhesins and fimbriae (*afr2*, *lifA*, *ral*, *paa*, etc.) [5,6,7,8,10]. All of the EPEC isolates (*n* = 40) studied in this work were also positive at least to one other colonization factor, and although different virulence profiles have been found, the one positive to *liftA*, *ral*, and *paa* (*n* = 28) was more frequently found, while isolates positives for *afr2* were scarce (*n* = 6). These results are in contrast with those described by Dow et al. [10] in Central Europe, who found a high degree of *afr2* positive EPEC pathogenic strains, while *lifA* was rarely found. This can reflect the complexity to also define the pathogenic capacity of a strain by genotypic traits.

Concerning *Eimeria* spp., 70% of growing rabbits and 30% of preweaning rabbits were positive, mostly in coinfection with *C. spiroforme*, EPEC, or rotavirus A (Figure 2). These results are in agreement with previous studies [8,24] where *Eimeria* spp. were found to be involved in rabbit enteropathies. Up to 11 *Eimeria* species have been described as specific pathogens for rabbits, with different degrees of pathogenicity among them [8]. Although Garcia-Rubio and colleagues [24] described a trend in co-infection between *Eimeria* spp. and Gram-negatives, we did not observe such a tendency but saw more co-infection with *C. spiroforme*. The fact that we detected the *eae* gene, and therefore EPEC, and *C. spiroforme* by qPCR directly from samples and they cultured the bacteria for detection can explain these discrepancies. Furthermore, this study did not distinguish between the 11 different *Eimeria* spp.; thus, it was not possible to identify whether the probability of coinfection with different bacteria varies depending the species of *Eimeria* present in the digestive track.

Although rotavirus is considered only mildly pathogenic, it has been hypothesized that, under field conditions, rotavirus seldom exerts direct pathogenic activity, and more frequently, it triggers the development of other infections. It primarily causes damage on the mucosa, thus predisposing the attachment and replication of bacteria [13]. A higher presence of rotavirus A in sick rabbits than in healthy ones was detected by Domingo et al. [25], providing epidemiological evidence of rotavirus A pathogenicity in this animal species. In the present study, rotavirus A was detected in 44% of the growing rabbits analyzed (Figure 3), usually coinfecting with other pathogens, being identified far less often in the two other age groups. The presence of maternal antibodies until 30 to 45 days post birth in rabbits has been related to the protection against this virus in young animals [26], which can explain the differences found between the different groups.

Some surveys on enteric rabbit disease have shown that 99% of *C. perfringens* isolates are toxin type A [27], but their involvement as causative agents is questionable [18]. Some studies have shown that *C. perfringens* type A rabbit strains which produce the Beta 2 toxin have higher pathogenic activity in vitro [28], and a study by Garcia et al. [14] also showed that *C. perfringens* enterotoxin can damage rabbit colonic loops, which can be used as an animal model, but so far, any attempts to experimentally reproduce enterotoxaemia by *C. perfringens* in rabbits have failed [18]. In our study, *C. perfringens* was detected at very low frequencies in all ages studied and almost always coinfected the digestive track with other agents, thus indicating that *C. perfringens* might not be an important pathogen in rabbits.

Recently, enterotoxigenic *B. fragilis* was described as a potential pathogen in preweaning rabbits. Watery diarrhea and high mortality not responding to antibiotic treatment while only controlled by the use of autogenous vaccine are the main features associated with its presence [16]. In this study, most of the positive cases were found in the preweaning age group (Figure 1) with 36% of the cases analyzed for enterotoxigenic *B. fragilis* being positive. Although the pathogenicity of *B. fragilis* has only been experimentally confirmed in newborn rabbits [29], some rabbit microbiome studies have observed an increase of *B. fragilis* in rabbits affected by enteropathies when compared to healthy rabbits [30]. Its real implication in rabbit digestive disorders has not yet been fully understood, and further research is needed to attribute the primary role in rabbit enteropathies.

*Salmonella* spp. is an important pathogen of high concern, in terms of economic damage, and it is considered as a primary pathogen in rabbits [31]. Although previous studies have shown that salmonellosis increased in Spain and Italy during the last decade of the past century [12,31], we only detected nine cases out of 757 total cases in the present work (Table 2). This can be attributed to the fact that, even if we routinely diagnose *Salmonella* spp. in samples coming from rabbit farms, most of the samples are sent framed in a scenario of sepsis in young rabbits or metritis in does. As this work includes exclusively cases where digestive disorders were described, other clinical signs such as the ones described above were excluded from the study. Thus, the unexpected low percentage of *Salmonella* spp. found may be attributed to the design of the study. Nevertheless, *Salmonella* spp. remains an important pathogen, because salmonellosis, albeit being a sporadic disease, usually produces high morbidity and mortality rates in affected rabbitries [18,32].

*Enterococcus hirae* has only been detected in two cases in young rabbits (<15 days old) (Table 2) and is associated with diarrhea in the first week of age and wasting syndrome and hypotrichosis in the following weeks. This syndrome has been previously described in rabbits associated with this etiological agent in Spain. In *E. hirae* infections, no significant lesions of inflammation are observed by histopathology in the intestine, but they attach to the brush border of enterocytes and form small masses of bacteria in the intestinal lumen, thus leading to a malabsorption syndrome, which explains the subsequent clinical signs observed [15]. Nevertheless, as it has been shown in this work, this process appears only sporadically in commercial rabbit farms.

Epizootic Rabbit Enteropathy (ERE) is a severe gastrointestinal syndrome that was first described in French intensive rabbit farms in 1997, which mainly affects post-weaning rabbits, causing high mortality rates in the absence of medication, but its etiology is still not completely understood [8], and its diagnosis cannot be established by laboratorial tests. Recent microbiota characterization studies using molecular approaches and modern sequencing techniques indicate that ERE could be caused by diet alterations or stress given by a wrong driving into the farm [30,33]. Additionally, ERE has been related to the proliferation of a new *Clostridium* species, *Clostridium cuniculi* [34], although this relation has not been experimentally validated yet. We cannot exclude that many of the cases studied in growing rabbits are related to this severe disease, but we cannot confirm those cases as ERE either.

Concerning wild rabbits, the main pathogens of the digestive system described in Spain are parasites [35]. The presence of wild rabbits near farms is common in the Peninsula, although biosecurity measures often prevent contact. However, we would find a comparative study of microorganisms that cause gastroenteric processes in wild rabbits interesting. 

## 5. Conclusions

Our study provides an overview of the etiological agents present in rabbit enteric diseases in the Iberian Peninsula during 2018–2019. From the high number of samples included in the study, we conclude that colibacillosis by EPEC is very important in young rabbits (<15 days old) as a single infectious disease; meanwhile, the older the rabbits are, the more complex the digestive diseases become. In preweaning animals EPEC remains as an important pathogen, but other pathogens grow in importance, for example, *C. spiroforme* and enterotoxigenic *B. fragilis* are usual co-infections with EPEC and *C. spiroforme* and, less frequently, with *Eimeria* spp. Concerning growing rabbits, we cannot find any preeminent pathogen over the rest, finding a large variety of coinfections among *C. spiroforme*, *Eimeria* spp., EPEC, and Rotavirus A and thus showing a much more complex scenario where a single cause for the disease cannot be attributed.

## Figures and Tables

**Table 1:** Clinical cases studied by different techniques presented in an age distributed format, from January 2018 to June 2019.

Technique Used	Young Rabbits	Preweaning Rabbis	Growing Rabbits	Total Cases Studied
Microbiological culture	95	117	545	757
EPEC ^1^ qPCR ^2^	95	117	545	757
*C. spiroforme* qPCR	95	117	545	757
*C. perfringens* qPCR	95	117	545	757
Rotavirus A qPCR	95	117	545	757
*Eimeria* spp. qPCR	59	77	406	542
*B. fragilis*^3^ qPCR	35	64	251	349

^1^ EPEC: Enteropathogenic *Escherichia coli.* ^2^ Real time polimerase chain reaction ^3^ Enterotoxigenic *Bacteroides fragilis*.

**Table 2:** Etiological agent, gene target, and coded protein identified by the qPCR kits used.

Etiological Agent	Gene Target	Coded Protein
EPEC	*eae*	Intimin
*C. spiroforme*	*sbs*	CSTb ^1^
*C. perfringens*	*cpa (plc)*	Alpha toxin
Rotavirus A	*gene7*	NSP3 ^2^
Enterotoxigenic *B. fragilis*	*bftp*	enterotoxin
*Eimeria* spp.	*16S*	D16S rRNA

^1^*C. spiroforme* toxin binding component; ^2^ non-structural protein3.

**Table 3:** Pathogens detected by bacteriological culture in age distributed cases.

Isolated Agent	Young Rabbits (<15 days old)	Preweaning Rabbits	Growing Rabbits	Total Positive Cases (%)
*Salmonella* spp.	2/95	5/117	2/545	9/757 (1.19%)
*E. hirae*	2/95	0/117	0/545	2/757 (0.26%)

**Table 4:** Genetic characteristics of the *E. coli* isolates studied.

Genetic Attributes					Number of Isolates
*eae*	*afr2*	*liftA*	*ral*	*paa*	
-	-	-	-	-	38
+	-	+	+	+	28
+	-	+	-	-	4
+	-	+	+	-	1
+	-	-	+	+	1
+	+	-	-	+	5
+	+	-	-	-	1
